# p53 Regulates a miRNA-Fructose Transporter Axis in Brown Adipose Tissue Under Fasting

**DOI:** 10.3389/fgene.2022.913030

**Published:** 2022-06-06

**Authors:** Isabel Reinisch, Ingeborg Klymiuk, Helene Michenthaler, Elisabeth Moyschewitz, Markus Galhuber, Jelena Krstic, Magnus Domingo, Fangrong Zhang, Michael Karbiener, Nemanja Vujić, Dagmar Kratky, Renate Schreiber, Michael Schupp, Georgia Lenihan-Geels, Tim J. Schulz, Roland Malli, Tobias Madl, Andreas Prokesch

**Affiliations:** ^1^ Gottfried Schatz Research Center for Cell Signaling, Metabolism and Aging, Division of Cell Biology, Histology and Embryology, Medical University of Graz, Graz, Austria; ^2^ Gottfried Schatz Research Center for Cell Signaling, Metabolism and Aging, Division of Molecular Biology and Biochemistry, Medical University of Graz, Graz, Austria; ^3^ Key Laboratory of Gastrointestinal Cancer, Ministry of Education, School of Basic Medical Sciences, Fujian Medical University, Fuzhou, China; ^4^ Global Pathogen Safety, Takeda Austria Manufacturing AG, Austria; ^5^ Institute of Molecular Biosciences, University of Graz, NAWI Graz, Graz, Austria; ^6^ BioHealth Graz, Graz, Austria; ^7^ Cardiovascular Metabolic Renal (CMR)- Research Center, Institute of Pharmacology, Charité-Universitätsmedizin Berlin, Corporate Member of Freie Universität Berlin, Humboldt- Universität zu Berlin, Berlin, Germany; ^8^ Department of Adipocyte Development and Nutrition, German Institute of Human Nutrition Potsdam-Rehbrücke, Nuthetal, Germany

**Keywords:** p53, metabolism, fasting, brown adipose tissue, miRNA, fructose

## Abstract

Active thermogenic adipocytes avidly consume energy substrates like fatty acids and glucose to maintain body temperature upon cold exposure. Despite strong evidence for the involvement of brown adipose tissue (BAT) in controlling systemic energy homeostasis upon nutrient excess, it is unclear how the activity of brown adipocytes is regulated in times of nutrient scarcity. Therefore, this study aimed to scrutinize factors that modulate BAT activity to balance thermogenic and energetic needs upon simultaneous fasting and cold stress. For an unbiased view, we performed transcriptomic and miRNA sequencing analyses of BAT from acutely fasted (24 h) mice under mild cold exposure. Combining these data with in-depth bioinformatic analyses and *in vitro* gain-of-function experiments, we define a previously undescribed axis of p53 inducing miR-92a-1-5p transcription that is highly upregulated by fasting in thermogenic adipocytes. p53, a fasting-responsive transcription factor, was previously shown to control genes involved in the thermogenic program and miR-92a-1-5p was found to negatively correlate with human BAT activity. Here, we identify fructose transporter *Slc2a5* as one direct downstream target of this axis and show that fructose can be taken up by and metabolized in brown adipocytes. In sum, this study delineates a fasting-induced pathway involving p53 that transactivates miR-92a-1-5p, which in turn decreases *Slc2a5* expression, and suggests fructose as an energy substrate in thermogenic adipocytes.

## Introduction

Cold-activated brown adipocytes are specialized cells that dissipate chemical energy to drive non-shivering thermogenesis (NST), thereby acting as a “metabolic sink” by taking up large amounts of glucose (Klepac et al., 2019) and fatty acids (Townsend and Tseng, 2014) from the circulation. The thermogenic functions of brown adipose tissue (BAT) are largely mediated by uncoupling the proton gradient from the electron transport chain *via* uncoupling protein 1 (UCP1) to generate heat instead of ATP (Nedergaard et al., 1977). NST is mainly driven by cold-induced activation of the sympathetic nervous system, which activates a downstream cascade that culminates in the activation of UCP1 (Harms and Seale, 2013). Recent advances in the field have delineated a set of peptides, signaling molecules, and hormones that influence the activity of brown adipocytes independently of or in addition to an increased sympathetic drive (Li et al., 2019). Interest in the search for BAT activators was spurred by the description of the central role of BAT in controlling whole-body energy homeostasis and metabolic health (Bartelt and Heeren, 2014).

The first evidence, coupling energy expenditure in BAT with nutrient intake, came from studies suggesting that BAT is activated upon caloric excess to counteract the effects of chronic overeating (Rothwell and Stock, 1997). Therefore, studies on thermogenic adipocytes have focused on finding ways to exploit the energy-dissipating properties of BAT as a pharmacological target to treat metabolic disease (Harms and Seale, 2013). However, reports on the functions and activities of BAT in nutrient shortage are currently limited (Reinisch et al., 2020).

From the viewpoint of a thermogenic cell, fasting and cold stress seem to be two processes with opposing biochemical demands. Whereas thermogenesis depends on effective utilization of energy substrates, upon nutrient deprivation, energy substrates (especially glucose) need to be conserved to be directed to the brain. Additionally, several fasting-induced metabolic pathways and signaling molecules, such as β-adrenergic signaling, fatty acids, ketone bodies, and fibroblast growth factor 21 (Fgf21), are well-known inducers of BAT activity (Reinisch et al., 2020). Therefore, counterregulatory mechanisms that balance preservative processes under fasting with the catabolic drive under cold exposure must exist in thermogenic adipocytes.

This study aimed to define BAT activity upon nutrient deprivation with simultaneous mild cold exposure and to elucidate molecular mechanisms that balance thermogenic and energetic demands in BAT of mice. By using unbiased omics approaches, we examined transcriptome and microRNA (miRNA) signatures in BAT upon fasting in mildly cold-stressed mice. In combination with functional *in vitro* experiments, we identified a fasting-selective pathway in BAT involving transcription factor p53, the miRNA miR-92a-1-5p, and the fructose transporter *Slc2a5*, implying a regulated, nutrient-dependent uptake of fructose as an energy substrate in brown adipocytes.

## Results

### Acute Fasting Mediates Major Alterations in BAT of Mildly Cold-Stressed Mice

**FIGURE 1:**
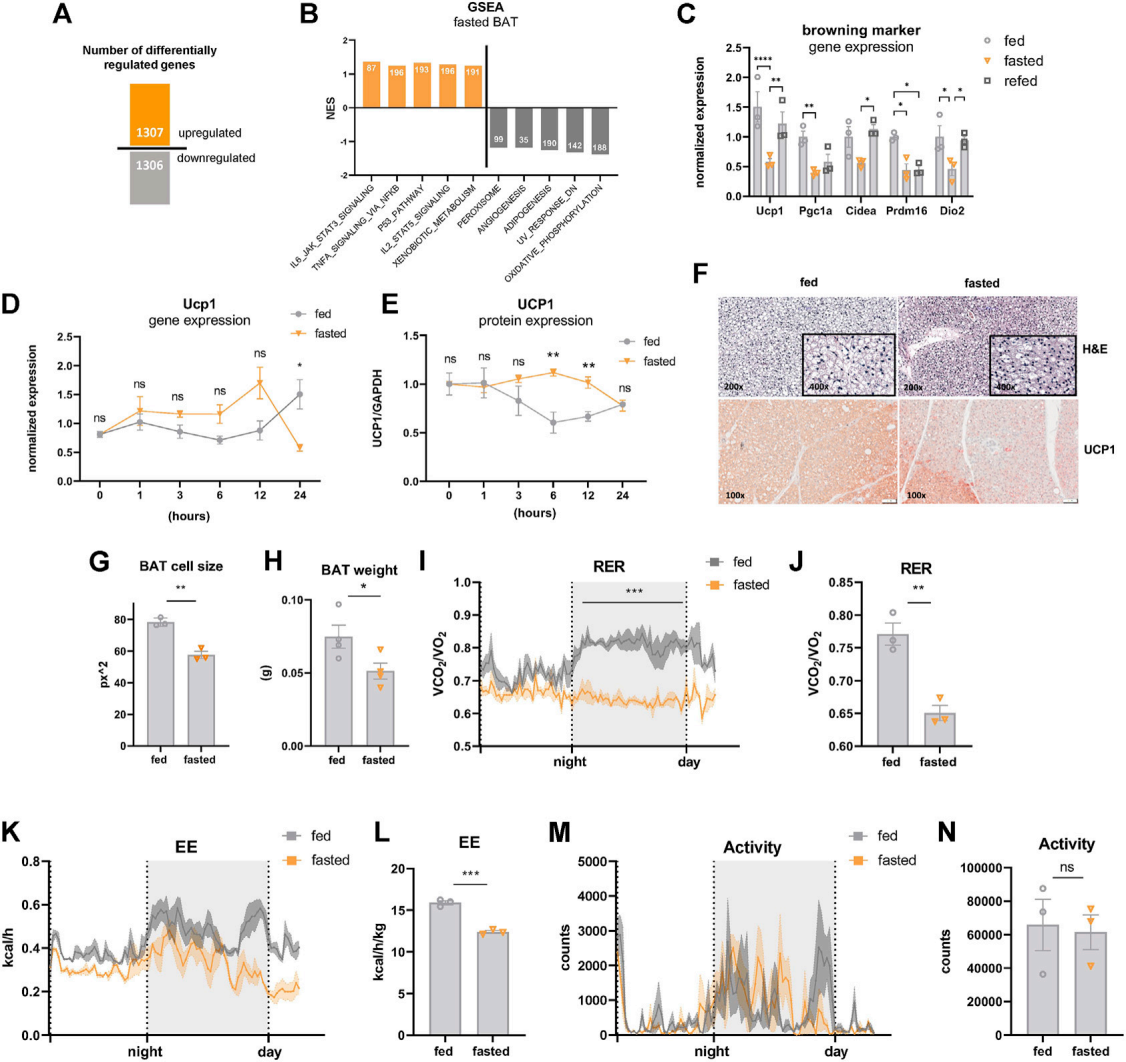
Acute fasting-mediated alterations in BAT of mildly cold-stressed mice. **(A)** Number of up- and downregulated (1.5 x, FDR5) genes in BAT of 24 h fasted mice. **(B)** GSEA Hallmark analysis results with top five pathways with highest or lowest normalized enrichment scores (NES). Numbers of mapped genes are shown in the bars. **(C)** BAT mRNA expression of genes encoding for browning markers of ctrl, 24 h fasted, and 4 h refed mice. **(D)** *Ucp1* mRNA and **(E)** UCP1 protein expression (from WES digital western blotting) in BAT harvested after 1, 3, 6, 12, and 24 h of fasting with samples from circadian-matched *ad libitum* fed controls. GAPDH served as loading control. **(F)** Representative images (magnification, ×100) of BAT of ctrl and 24 h fasted mice stained with hematoxylin and eosin **(E–H)** and immunohistochemistry with anti-UCP1 antibody. **(G)** Cell size of was analyzed by using ImageJ and indicated as square pixels (px^2^). **(H)** BAT weight in grams (g) of ctrl and 24 h fasted mice. **(I,J)** Respiratory exchange ratio (RER), **(K,L)** energy expenditure (EE), and **(M,N)** motility of ctrl and 24 h fasted mice as validated by indirect calorimetry in metabolic cages. Data are presented as mean values ± SEM. Significant differences in the mRNA expression of fed, fasted, and refed mice were analyzed by 2-way ANOVA and Tukey’s multiple comparisons test, for BAT weight, RER and energy expenditure unpaired t*-*test was performed. Differences not indicated with asterisks or indicated with ns are not statistically significant (*p* > 0.05). ***p < 0.001, **p < 0.01, and *p < 0.05.

The elaborate metabolic response to nutrient deprivation is achieved through a fine-tuned transcriptional program, which is tissue-specific and dependent on the duration and frequency of the fasting stimulus (de Cabo and Mattson, 2019). Due to the lack of information about the transcriptional signature of BAT upon fasting, we performed mRNA transcriptome analysis of male C57BL/6J mice fasted for 24 h or *ad libitum* chow diet-fed controls and maintained under mild cold stress (22°C, singly housed in grid bottom cages). It is clearly established that room temperature is below thermoneutrality in mice (∼30°C), which is compensated by a substantial increase in the thermogenic properties of BAT (Ganeshan and Chawla, 2017). Under these conditions, 1,307 genes were upregulated and 1,306 genes were downregulated in BAT of fasted vs. control fed mice (Figure 1A), illustrating a striking remodeling of the BAT transcriptomic landscape upon fasting. Performing gene set enrichment analysis revealed a shift towards downregulation of genes in pathways closely associated with the thermogenic program: Oxidative phosphorylation, adipogenesis, and angiogenesis were among the top-downregulated metabolic pathways under acute fasting (Figure 1B). Decreased oxidative phosphorylation is consistent with reduced fasting mRNA levels of Pgc1α (Figure 1C), the master regulator of mitochondrial biogenesis (Wu et al., 1999). In line with these results, the expression of BAT-selective genes was significantly downregulated after 24 h of fasting (Figure 1C). Four hours of refeeding reversed the fasting-mediated decrease in the expression of most thermogenic genes. To shed light on the immediate-early starvation response, BAT samples were harvested from mice maintained at room temperature after 1, 3, 6, 12, or 24 h of food withdrawal. To control for circadian rhythmicity of BAT activity (Gerhart-Hines et al., 2013), control samples were harvested at the same time points from mice fed *ad libitum* with chow diet. Our data showed that *Ucp1* mRNA expression followed a different rhythmicity in the fed compared to the fasted group (Figure 1D). *Ucp1* expression levels increased after the first 12 h of fasting, but were significantly decreased after 24 h of fasting compared to *ad libitum* controls. Similar to transcript levels, we found that UCP1 protein levels were also significantly increased after 6 and 12 h of nutrient deprivation and decreased to control levels after 24 h of fasting (Figure 1E). Furthermore, brown adipocyte cell size was decreased (Figures 1F,G), UCP1 immunostaining was diminished (Figure 1F), and BAT weight was reduced (Figure 1H) in 24 h fasted mice compared to fed control mice. Systemically, the respiratory exchange ratio (RER), as an indicator of whole-body fuel utilization, was significantly reduced during fasting (Figures 1I,J), coinciding with a marked decrease in energy expenditure (Figures 1K,L). In situations of severe cold and/or prolonged fasting, mice can enter torpor, which is a hibernation-like state characterized by strongly reduced locomotion, a slowdown in metabolism, and a body temperature below 32°C (Geiser et al., 2014). However, we did not detect any signs of torpor, as indicated by unchanged activity of the mice upon 24 h of fasting in mild cold (Figures 1M, N). These data suggest that BAT activity of mice under mild cold stress gradually increases in the first hours after nutrient withdrawal, but is dampened upon 24 h of fasting.

### p53 Signaling Is Activated by Acute Fasting in Brown Adipocytes

**FIGURE 2:**
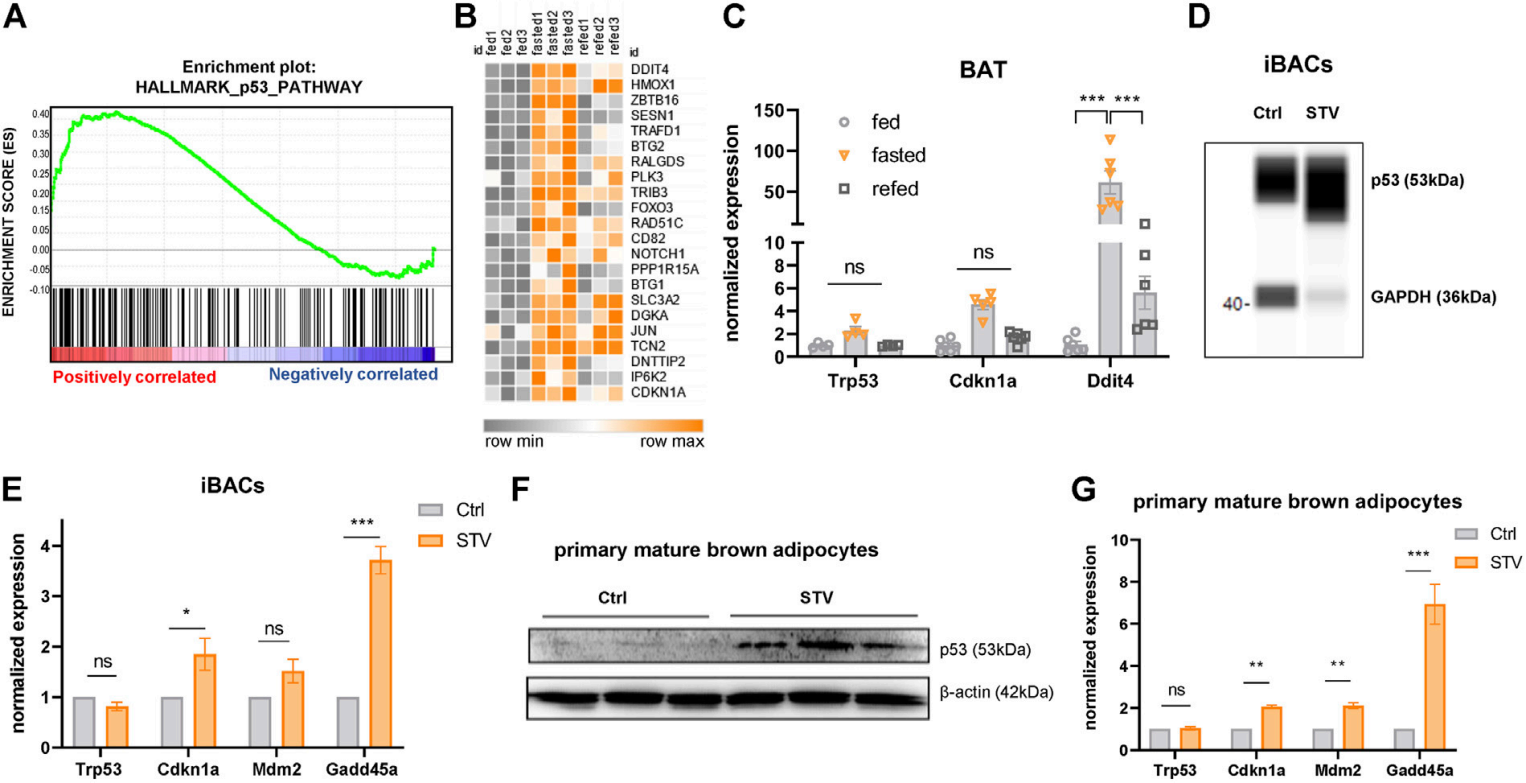
p53 signaling is activated by acute fasting in brown adipocytes. **(A)** Gene set enrichment blot of 193 genes annotated as p53 pathway (NES = 1.33, nominal *p*-value = 0.00) in BAT of 24 h fasted versus fed mice. **(B)** Heatmap showing p53 target genes differentially regulated in BAT of control fed, 24 h fasted and 4 h refed mice. **(C)** mRNA expression of p53 target genes in BAT of ctrl, 24 h fasted, and refed mice as validated by qPCR. **(D)** WES digital western blot of p53 protein of differentiated iBACs cultured in growth medium (Ctrl) or starvation medium (STV) for 24 h. GAPDH served as a loading control. **(E)** mRNA expression of p53 target genes in differentiated iBACs in Ctrl or STV conditions. **(F)** Western blot of p53 in primary mature brown adipocytes in Ctrl or STV conditions. *β*-actin served as loading control. **(G)** mRNA expression of p53 target genes in primary mature brown adipocytes in Ctrl or STV conditions. Mean values ± SEM are shown, significant differences in the mRNA expression of fed, fasted, and refed mice were analyzed by 2-way ANOVA and Tukey’s multiple comparisons test. For *in vitro* data, unpaired t-test was performed. Differences not indicated with asterisks or indicated with ns are not statistically significant (*p* > 0.05). ***p < 0.001, **p < 0.01, and *p < 0.05.

The transcriptional landscape upon fasting is tightly regulated by a network of distinct nutrient-responsive transcription factors (Schupp et al., 2013; Goldstein et al., 2017). p53 was recently added to the list of fasting-related transcription factors due to its implications in the maintenance of energy homeostasis in the liver of fasted mice (Goldstein and Hager, 2015; Prokesch et al., 2017; Gonzalez-Rellan et al., 2021). In agreement with these reports, we found that p53 signaling was among the top-five upregulated pathways under fasting conditions in BAT of mildly cold-stressed mice (Figures 1B and, 2A, B). We confirmed the upregulation of the p53 target genes *Cdkn1a* and *Ddit4* in fasted BAT by qPCR (Figure 2C). The fasting-mediated increase in the expression of p53 target genes was abrogated by 4 h of refeeding (Figure 2C), indicating that the p53 activation is dependent on nutritional state. To test if the fasting-triggered increase in p53 signaling is a brown adipocyte-autonomous mechanism, we used two *in vitro* models: immortalized brown adipocytes (iBACs) and primary preadipocytes isolated and differentiated from the stromal vascular fraction of BAT. In line with the *in vivo* data, p53 protein expression and the transcript levels of p53 target genes were induced in mature iBACs (Figures 2D,E) and primary brown adipocytes (Figures 2F,G) after 24 h in starvation medium (STV). These data indicate that, also in brown adipocytes, p53 is a fasting-selective transcription factor that might be involved in the regulation of the switch from anabolic to catabolic processes upon nutrient deprivation.

### miR-92a-1-5p, a Previously Described Negative Marker for BAT Activity, Is Upregulated in Fasted BAT

**FIGURE 3:**
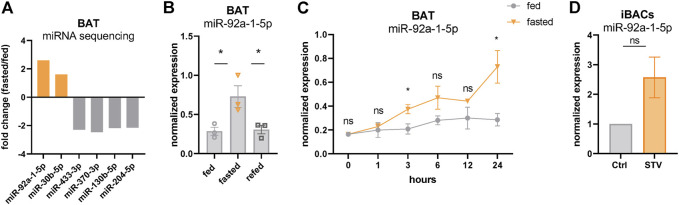
miR-92a-1-5p is upregulated in fasted BAT. **(A)** Top up- and downregulated miRNAs in 24 h fasted BAT determined by miRNA sequencing (*n* = 3). **(B)** Expression of miR-92a-1-5p in BAT of ctrl, 24 h fasted, and refed mice validated by qPCR. **(C)** Time-series of miR-92a-1-5p expression in BAT harvested after 1, 3, 6, 12, and 24 h of fasting in mildly cold-stressed mice. As control, BAT was harvested from *ad libitum* fed mice at the respective circadian-matched time points (*n* = 3). **(D)** Expression of miR-92a-1-5p in differentiated iBACs cultivated in growth medium or starved for 24 h (*n* = 3). Mean values ± SEM are shown, significant differences in the mRNA expression of fed, fasted and refed mice were analyzed by 2-way ANOVA and Tukey’s multiple comparisons test. For *in vitro* data, unpaired t-test was performed. Differences not indicated with asterisks or indicated with ns are not statistically significant (*p* > 0.05). *p < 0.05.

Our data showing p53 activation with concomitantly decreased BAT activity after 24 h of fasting is consistent with a recent study that demonstrated p53 as a negative regulator of the thermogenic program in brown adipocytes in mice (Zhao et al., 2020). Therefore, we further investigated the potential underlying regulatory mechanisms. Evidence about p53 acting as a direct transcriptional repressor is very scarce. Indeed, a meta-analysis has suggested that p53 acts solely as a transcriptional activator (Fischer, 2017), whereas most of the repressive effects observed upon p53 signaling activation may be indirect. We reasoned that these indirect effects might be a consequence of p53-mediated expression of transcriptional co-repressors or of miRNAs. To evaluate a possible contribution of miRNAs, we performed miRNA sequencing from the same BAT samples used for transcriptomics. miR-92a-1-5p, which has been previously described as a negative marker for BAT activity (Chen et al., 2016), emerged as the top upregulated miRNA in fasted BAT, whereas the expression of most other miRNAs was reduced (Figure 3A). qPCR verified the increased expression of miR-92a-1-5p in fasted BAT (Figure 3B) which returned to control levels after 4 h of refeeding, reflecting the nutrient-dependent signatures of p53 target genes (Figures 2B,C). More detailed analyses of fasting kinetics showed that mRNA expression levels of miR-92a-1-5p were immediately and increasingly induced by fasting, as validated in our time-course fasting experiment (Figure 3C). Finally, we confirmed the upregulation of miR-92a-1-5p in iBACs exposed to the starvation medium for 24 h (Figure 3D), indicating a brown adipocyte-autonomous mechanism of miR-92a-1-5p upregulation. Taken together, we delineated the miRNA profile of fasted BAT and identified miR-92a-1-5p as the top fasting-induced miRNA.

### Fasting-Induced miR-92a-1-5p Downregulates the Nutrient-Regulated Fructose Transporter *Slc2a5* in Brown Adipocytes

**FIGURE 4:**
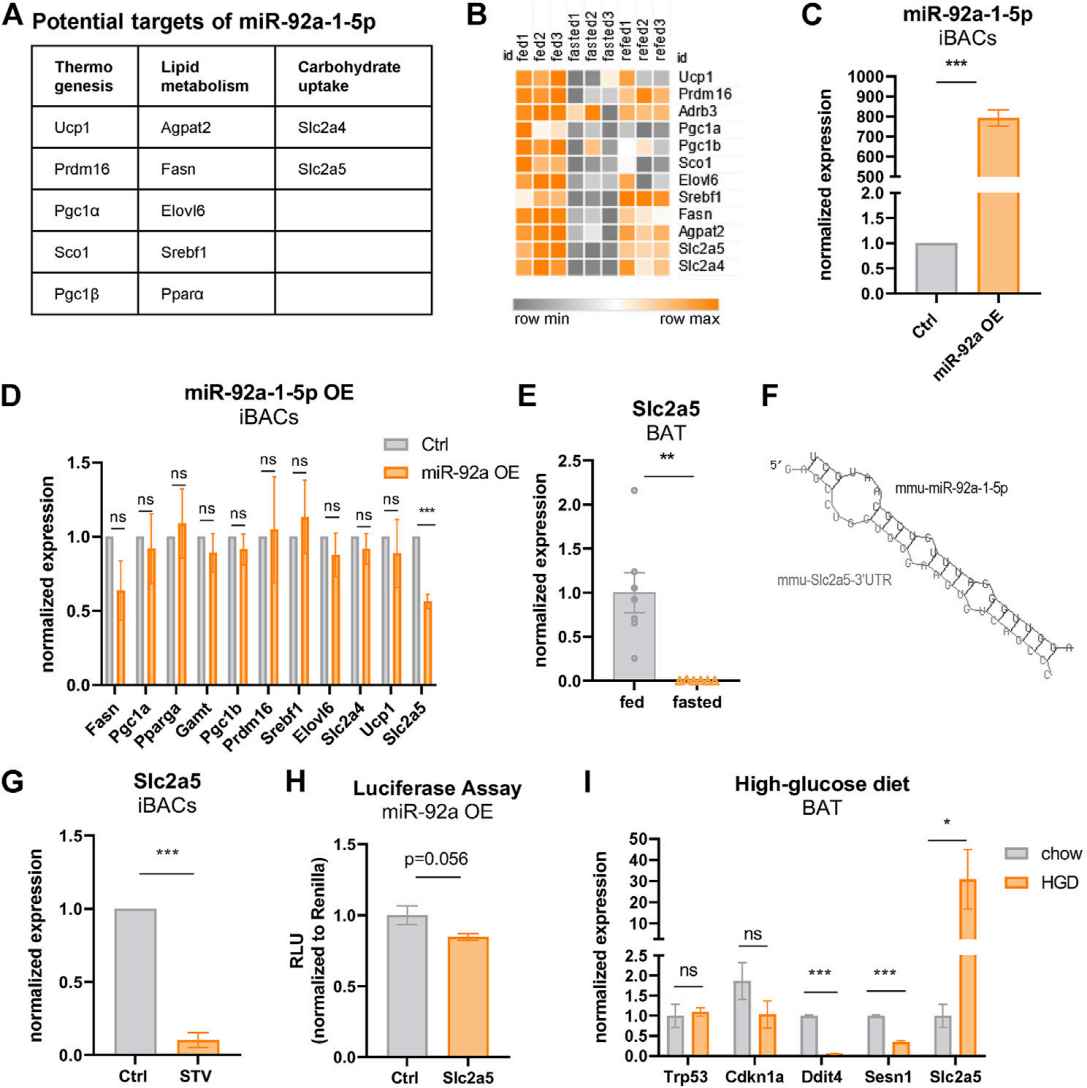
Fasting-induced miR-92a-1-5p downregulates the fructose transporter *Slc2a5* in brown adipocytes. **(A)** Predicted target genes of miR-92a-1-5p downregulated by fasting in BAT. **(B)** Heatmap of predicted, fasting-downregulated miR-92a-1-5p targets in BAT of ctrl, 24 h fasted, and refed mice. **(C)** qPCR expression levels of miR-92a-1-5p in iBACs after transfection with miRNA-mimic-92a-1-5p or non-targeting control. **(D)** mRNA expression of predicted target genes of miR92a-1-5p in iBACs after transfection of miR-mimc-92a-1-5p or non-targeting control. **(E)** mRNA expression of *Slc2a5* in BAT of fed or 24 h fasted mice. **(F)** Putative miRNA seed match of miR-92a-1-5p within the 3’UTR of *Slc2a5*. **(G)** mRNA expression of *Slc2a5* in iBACs cultured in standard growth medium or starved for 24 h. **(H)** Luciferase assay was performed by co-transfecting a reporter vector containing the 3’UTR of *Slc2a5* harboring the predicted binding sites of miR-92a-1-5p, with miR-92a-1-5p mimic or non-targeting control in HEK293 cells. **(I)** mRNA expression of *Slc2a5* and p53 target genes in BAT of mice fed chow or high-glucose diet. Mean values ± SEM are shown, significant differences in the mRNA expression of fed and fasted mice were analyzed by performing an unpaired t-test. For *in vitro* data, unpaired t-test was performed. Differences not indicated with asterisks or indicated with ns are not statistically significant (*p* > 0.05). ***p < 0.001, **p < 0.01, and *p < 0.05.

Combining our transcriptomic analysis with seed match predictions in the 3′ untranslated regions (UTR), we generated a list of potential miR-92a-1-5p target genes (Figure 4A), that are downregulated by fasting (Figure 4B) and associated with thermogenesis, lipid metabolism, or carbohydrate uptake. To functionally validate these predictions, we analyzed alterations in the expression of these candidate genes upon overexpression of miR-92a-1-5p mimic in iBACs (Figure 4C). We found no overt differences in the expression of most candidate genes tested, except for the markedly reduced expression of fructose transporter *Slc2a5* in iBACs (Figure 4D). In our transcriptome data, *Slc2a5* was the most diminished gene upon fasting in BAT (19-fold downregulation, Figure 4E). The putative interaction between miR-92a-1-5p and the predicted miRNA recognition element in the *Slc2a5* 3′UTR is shown in Figure 4F. Furthermore, 24 h exposure of iBACs to starvation medium, conditions in which p53 protein was stabilized (Figure 2D) and miR-92a-1-5p expression increased (Figure 3D), led to a drastic decrease in *Slc2a5* expression (Figure 4G). To validate the direct interaction between miR-92a-1-5p and *Slc2a5*, we cloned the 3′UTR of *Slc2a5* containing the predicted seed matches of miR-92a-1-5p into a luciferase reporter vector and performed co-transfection with miR-92a-1-5p mimic or a non-targeting control in HEK293 cells. Co-transfection of miR-92a-1-5p mimic trended to reduce the luciferase activity (Figure 4H), suggesting a direct interaction of miR-92a-1-5p with the 3′UTR of *Slc2a5*, which may lead to subsequent degradation of *Slc2a5* mRNA. To probe if increased nutrient supply modulates the expression of *Slc2a5 in vivo*, we challenged C57BL/6 mice with a high-glucose diet. Interestingly, high-glucose feeding led to a substantial increase in the expression of *Slc2a5* in BAT (Figure 4I), with a concomitant decline in the expression of p53 target genes (Figure 4I). These data suggest *Slc2a5*, a highly nutrient-responsive gene in BAT, as a direct target of fasting-induced miR-92a-1-5p. Moreover, we demonstrated that the expression of *Slc2a5* in BAT is induced by high-glucose feeding *in vivo*, corroborating a potential functional role for fructose metabolism in BAT.

### Fructose Is Taken up and Metabolized by Brown Adipocytes

**FIGURE 5:**
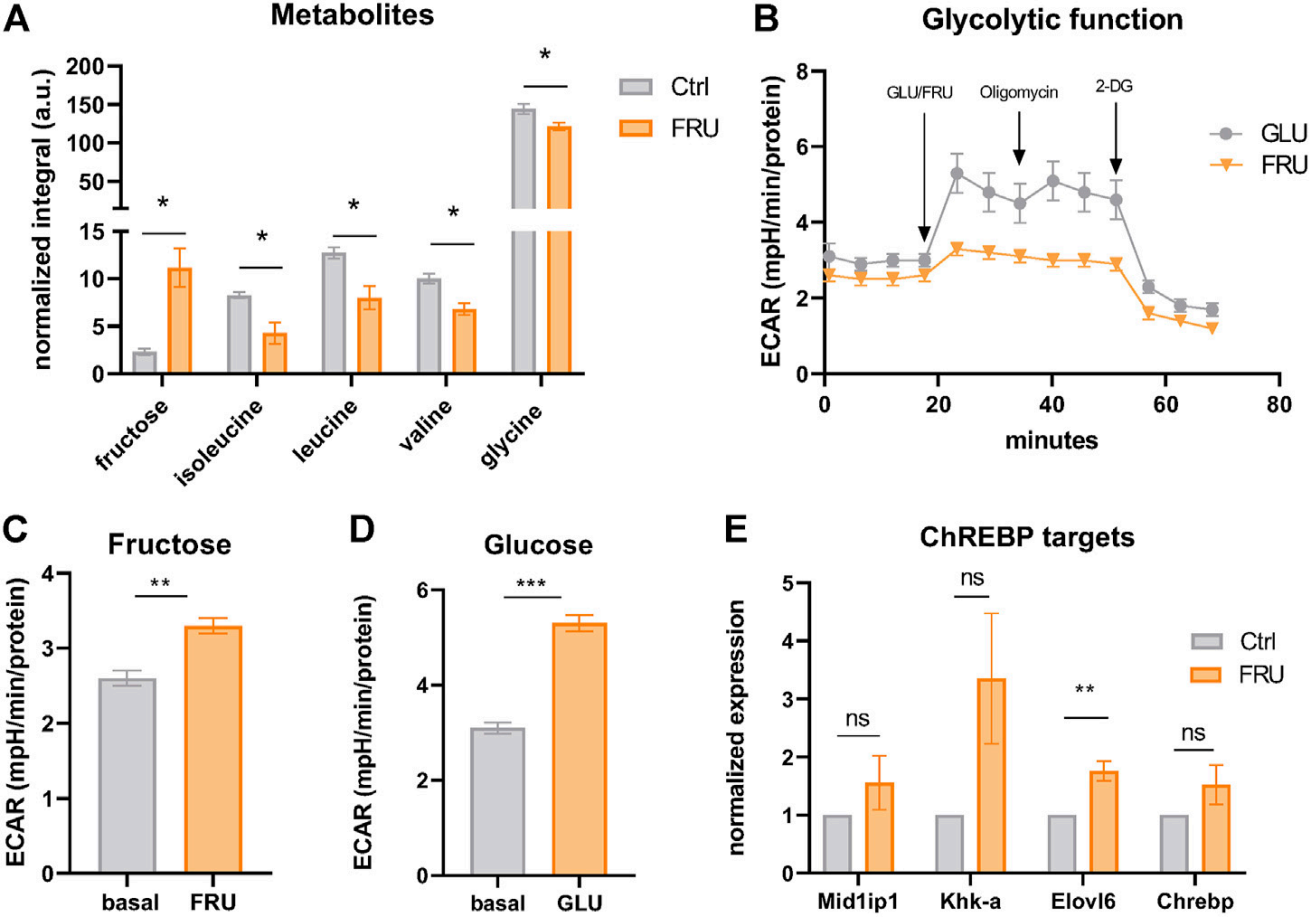
Fructose is taken up and metabolized by brown adipocytes. **(A)** Abundance of intracellular fructose and the amino acids isoleucine, leucine, valine, and glycine in iBACs cultivated in growth medium or supplemented with 5 g/L fructose measured by nuclear magnetic resonance (NMR). **(B)** Continuous extracellular acidification rate (ECAR) in BACs after the injection of 5 g/L fructose or glucose. Oligomycin and 2-deoxyglucose (2-DG) were acutely injected after measuring basal ECAR. **(C,D)** Basal ECAR (analyzed after 20 min of measurement) and stimulated ECAR after acute infusion of 5 g/L fructose **(C)** or glucose **(D)**. **(E)** mRNA expression of ChREBP target genes in iBACs kept in growth medium or supplemented with 5 g/L fructose. Mean values ± SEM are shown, significant differences in the mRNA expression of intracellular metabolites and seahorse experiments were analyzed by performing an unpaired t-test. For mRNA expression of ChREBP targets, unpaired t-test was performed. Differences not indicated with asterisks or indicated with ns are not statistically significant (*p* > 0.05). ***p < 0.001, **p < 0.01, and *p < 0.05.

SLC2A5 (also called GLUT5) is a member of the GLUT family of facilitated sugar transporters that, unlike the other members, has an almost exclusive affinity for fructose (Mueckler and Thorens, 2013). SLC2A5 is mainly expressed at the apical surface of intestinal epithelial cells, but also to a lower extent in testis, kidneys, brain, skeletal muscle, and adipose tissues (Cura and Carruthers, 2012). Previous studies have shown that fructose can be metabolized in an *in vitro* model of mature white adipocytes (Varma et al., 2015a; 2015b). However, the functional role of fructose in thermogenic adipocytes is unknown. To investigate whether fructose can be taken up by brown adipocytes, we supplemented the standard growth medium of iBACs with fructose and analyzed the abundance of intracellular metabolites by nuclear magnetic resonance metabolomics. Strikingly, fructose was avidly taken up by brown adipocytes, concomitant with a significant decrease in the abundance of branched-chain amino acids [BCAAs; isoleucine, leucine, valine, which are substrates for NST (Yoneshiro et al., 2019)] and glycine (Figure 5A). In white adipocytes, it was suggested that fructose is not catabolized *via* fructolysis but feeds into glycolysis after being phosphorylated at position six by hexokinase (Legeza et al., 2017). To probe if fructose is indeed metabolized via glycolysis, we quantified the extracellular acidification rate (ECAR) of iBACs after acute exposure to fructose or glucose in a Seahorse glycolytic function assay. Acute fructose infusion led to increased proton generation (or acidification), suggestive of conversion of fructose to lactate (Figures 5B,C), albeit to a lower extent than after glucose infusion (Figures 5B,D). Fructose is a potent inducer of signaling through the carbohydrate response element-binding protein (ChREBP), which controls the expression of genes involved in lipogenesis, glycolysis, and fructolysis (Ortega-Prieto and Postic, 2019). Analysis of ChREBP target genes in iBACs exposed to fructose revealed a trend toward increased expression of *Mid1ip1, Khk-a,* and *Chrebp-β*, and a significant induction of *Elovl6* (Figure 5E), which is critically involved in fatty acid elongation. Taken together, we demonstrated that fructose 1) can be taken up by brown adipocytes, 2) is, at least in part, directed into the glycolytic pathway, and 3) leads to partial activation of the carbohydrate-responsive ChREBP pathway. However, the metabolic pathways and consequences of fructose utilization in brown adipocytes requires further research.

### p53 Binds and Activates the miR-92a-1-5p Locus

**FIGURE 6:**
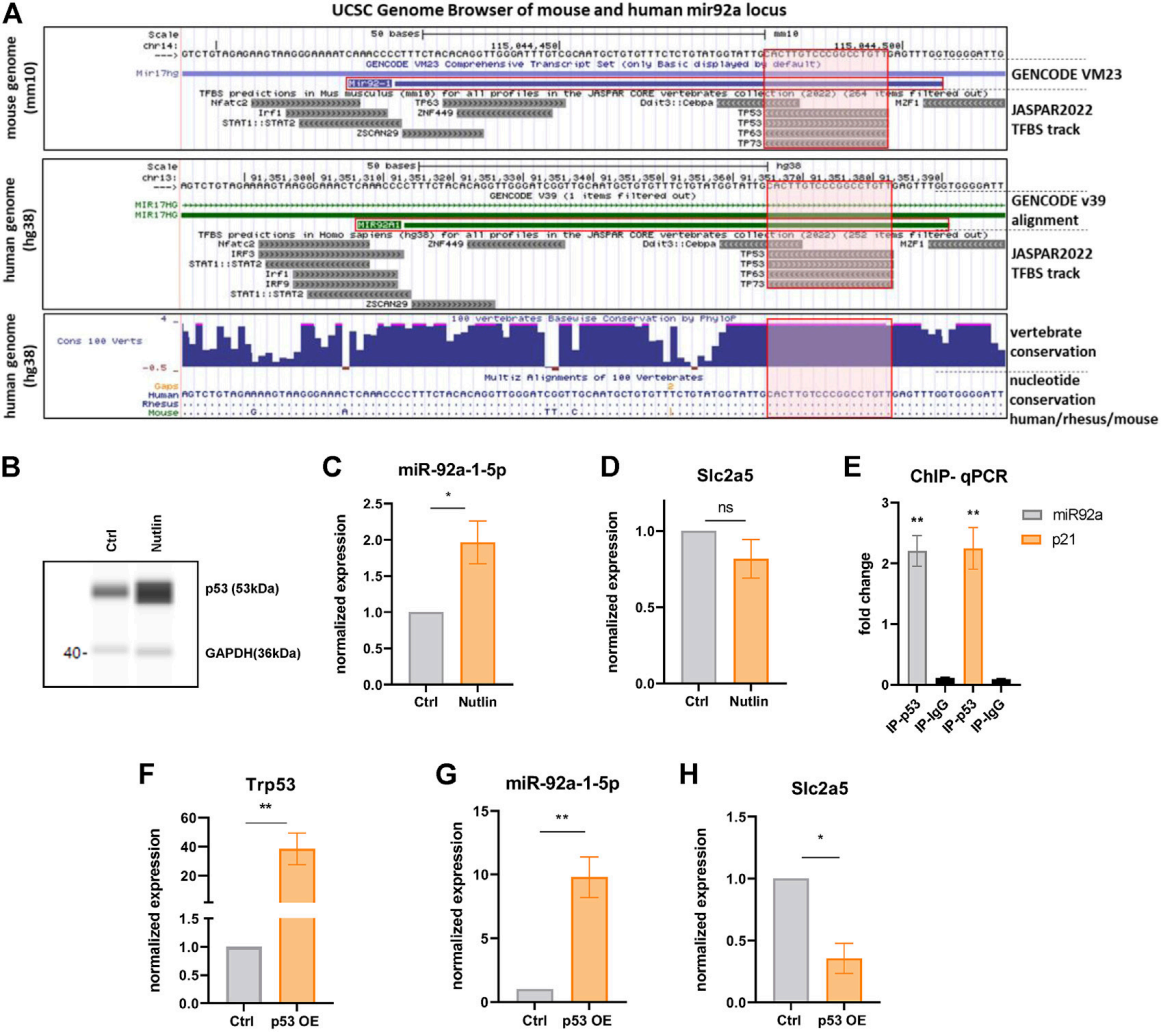
p53 binds and activates the miR-92a-1-5p locus. **(A)** Excerpt from the UCSC genome browser tracks from mouse (mm10) and human (hg38) genomes in the vicinity of the mir92a locus (Mir92-1 for mouse chromosome 14, MIR92A1 for human chromosome 13). Respective JASPAR tracks illustrate p53 binding motives (red shaded boxes) that are fully conserved between human, rhesus monkey, and mice (lowest box; dots depict sequence identity). **(B)** WES digital western blot of p53 protein of iBACs after nutlin or vehicle treatment. GAPDH served as a loading control. **(C)** miR-92a-1-5p and **(D)** *Slc2a5* expression after nutlin or vehicle treatment of iBACs. **(E)** Fold-enrichment of a p53-binding site at the miR-92a-1-5p locus. ChIP-qPCR was used to amplify chromatin derived from immunoprecipitations with anti-p53 antibody or anti-IgG antibody. A known p53 binding site in the *Cdkn1a* locus served as positive control and primer pairs targeting distant loci without predicted p53 binding sites served as negative control. **(F)** mRNA expression of *Trp53* upon overexpression of full length wild-type p53 (or transfection with empty control vector) in iBACs. Expression of **(G)** miR-92a-1-5p and **(H)** *Slc2a5* upon overexpression of p53 or control in iBACs. Data are presented as mean values ± SEM. Significances were determined by unpaired Student’s t-tests. Differences not indicated with asterisks or indicated with ns are not statistically significant (*p* > 0.05). *p < 0.05, and **p < 0.01.

**FIGURE 7:**
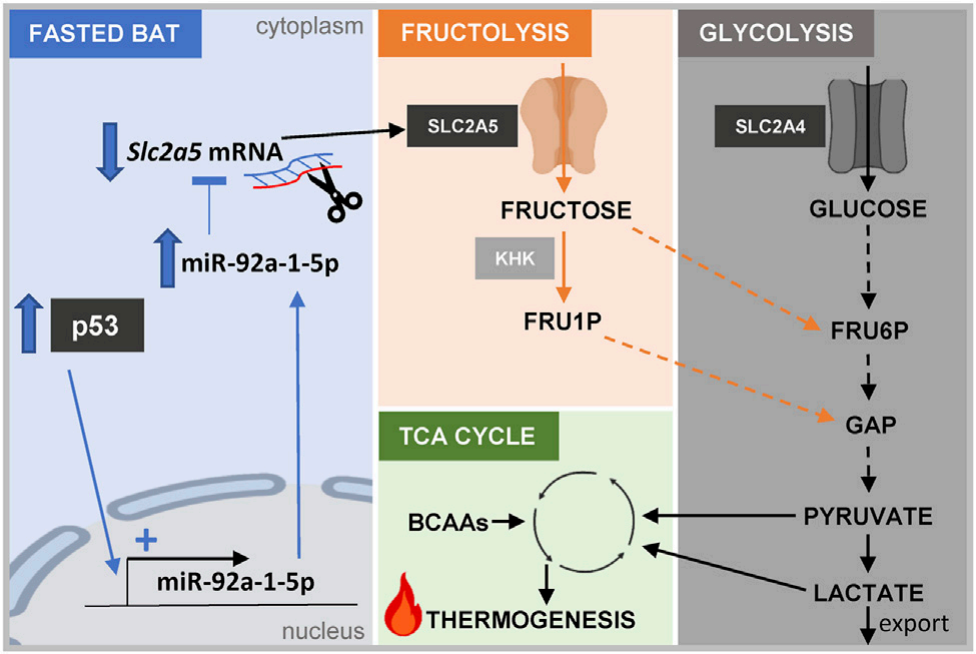
Graphical abstract. Scheme depicting the proposed connection between the p53/miR-92a-1-5p/Slc2a5 axis, fructose and glucose metabolism, and thermogenesis in brown adipocytes. Abbreviations: KHK, keto-hexokinase; FRU1P, fructose-1-phosphate; FRU6P, fructose-6-phosphate; GAP, glyceraldehye-3-phosphate; BCAAs, branched-chain amino acids.

Based on the function of p53 in regulating metabolism in response to various nutritional cues (Labuschagne et al., 2018) and on the co-regulation of miR-92a-1-5p and p53 targets in BAT (Figures 2C, 3B), we hypothesized that miR-92a-1-5p might be a direct nutrient-responsive target of p53. In line with this notion, JASPAR binding site predictions yield a p53 binding site in the mouse miR-92-1 locus (Figure 6A, red shaded boxes). Intriguingly, this p53 binding site is fully conserved in the human MIR92A1 locus (Figure 6A, lowest panel). To further examine if miR-92a-1-5p is a direct target of p53, we modulated the expression of p53 in mature iBACs. Pharmacologic stabilization and activation of p53 with the small molecule nutlin (Figure 6B) resulted in a significantly increased expression of miR-92a-1-5p (Figure 6C) and a tendency, albeit not significant, to downregulation of *Slc2a5* (Figure 6D). In addition, chromatin immunoprecipitation qPCR showed a significant enrichment of p53, over a negative control region and IgG antibody control, at the miR-92a-1-5p locus, confirming a direct binding of p53 to the miR-92 locus (Figure 6E). p53 binding in a known recognition element in the *Cdkn1a* (p21) locus served as a positive control (Figure 6E). In line with these findings, overexpression of full-length p53 (Figure 6F) increased the expression of miR-92a-1-5p (Figure 6G) with concomitant decrease of *Slc2a5* (Figure 6H). Taken together, p53 activation, followed by an increased miR-92a-1-5p abundance with concomitant downregulation of *Slc2a5* may represent a functional signaling cascade regulating fructose utilization in brown adipocytes with p53 as the nutrient-responsive upstream regulator (Figure 7).

## Discussion

In many peripheral tissues, nutrient deprivation elicits a tightly controlled switch from anabolic to energy-conserving mechanisms that provide energy substrates to the brain, which cannot adequately store energy or efficiently utilize fatty acids that are abundantly released during fasting (Secor and Carey, 2016). Little is known about the fasting response of BAT, especially during simultaneous cold exposure when demands for energy conservation theoretically counteract energy consumption for thermogenesis (Reinisch et al., 2020). We defined the transcriptomic signature of BAT from 24 h fasted mice exposed to mild cold stress at the mRNA and miRNA level. Our data suggest that within 24 h, maintenance of energy homeostasis is favored over thermogenesis. This was reflected in decreased expression of BAT-selective genes and genes involved in oxidative phosphorylation, diminished BAT weight, and reduced RER, as well as in disruption of diurnal mRNA and protein rhythmicity of Ucp1, that was recently shown to be under control of circadian and metabolic regulator Rev-erbα (Gerhart-Hines et al., 2013). Decreased oxidative phosphorylation was in line with a recent publication showing reduced mitochondrial content in BAT of mice that were intermittently fasted (Harney et al., 2021). In contrast, earlier studies showed no differences in norepinephrine turnover as a proxy for sympathetic drive, in the expression of thermogenic genes, or in BAT weight after 24 h of fasting in mildly cold stressed mice (Knehans and Romsos, 1983; Ding et al., 2016). These discrepant results may be attributed to different housing conditions of mice. The mice in our study were housed individually, without nesting material, and in grid-bottom cages to prevent coprophagy, eating of bedding material, and external thermo-homeostasis through nesting and shared body heat. Thus, the housing conditions in our study represent a more severe cold and fasting stress.

Previously described fasting-induced core regulators of the thermogenic program involve metabolites like fatty acids and ketone bodies, signaling pathways like norepinephrine signaling, and hormones like glucagon, glucocorticoids, and FGF21 (Reinisch et al., 2020). However, whereas fasting-selective transcription factors have been well-described in the liver (Goldstein and Hager, 2015), the transcriptional regulation of the thermogenic program upon fasting in adipocytes has received little attention. The transcription factor p53 was originally described as main player in cancer development, as evidenced by the high prevalence of *TP53* mutations in several cancer types in humans (Baker et al., 1990a; Baker et al., 1990b; Olivier et al., 2002). However, besides its vital function as tumor suppressor, more recent studies have established p53 as an important regulator of metabolism and tissue homeostasis in non-cancer contexts (Lacroix et al., 2020). This was corroborated by findings of our group, which demonstrated that the p53 pathway is activated within 24 h of nutrient withdrawal in the major fasting-responsive mouse tissues [white adipose tissue, liver, and skeletal muscle (Schupp et al., 2013)]. Moreover, p53 has been delineated as a nutrient-responsive transcription factor that is pivotal for the physiological response to fasting in the liver (Prokesch et al., 2017; Gonzalez-Rellan et al., 2021). In these studies, p53 activity was regulated through stabilization of the p53 protein by upstream stress stimuli, rather than through upregulation of p53 mRNA. This is consistent with our findings in BAT where p53 mRNA levels remained unchanged by fasting (Figure 2C), while p53 signaling was broadly activated (Figures 2A–C). Interestingly, earlier work in colon cancer cells showed p53 stabilization upon AMPK-mediated phosphorylation (Jones et al., 2005), a mechanism that could also play a role in brown adipocytes under fasting.

In adipocytes, there is increasing evidence for a function of p53 in the control of lipid metabolism, (brown) adipocyte differentiation, thermogenesis, and systemic energy homeostasis (Al-Massadi et al., 2016; Krstic et al., 2018). Whereas a previous report described p53 as a positive regulator of the thermogenic program (Al-Massadi et al., 2016), a more recent study demonstrated KMT5c-knock out-dependent p53 induction responsible for the diminished expression of genes in the thermogenic program (Zhao et al., 2020), highlighting the context-dependent mode of action of p53.

Thus, based on our data and previous reports, p53 seems to act as fasting-dependent negative regulator of the thermogenic program in BAT. However, since there is little evidence for p53 as a direct transcriptional repressor (Fischer, 2017), we hypothesized that the repressive actions of p53 in BAT might be regulated indirectly by miRNAs. While we cannot entirely rule out a direct impact of p53 on Slc2a5, our collective data including luciferase assay and miR-92a-1-5p overexpression, indicate that p53 acts through miR-92a-1-5p. In general, p53 regulates a plethora of different miRNAs in various contexts, either by inducing their transcription or by promoting their processing and maturation (Hermeking, 2012; Goeman et al., 2017). In addition, several BAT-derived miRNAs have been described to affect the thermogenic program (Goody and Pfeifer, 2019). Here, we elucidated miR-92a-1-5p as the top fasting-responsive miRNA in BAT. Intriguingly, Chen et al. (2016) *.* showed that miR-92a is present in human and mouse BAT-derived exosomes and that the abundance of miR-92a in serum exosomes inversely correlates with human BAT activity. Seed match prediction analysis revealed a plethora of thermogenesis-, lipid-, and carbohydrate-associated genes as potential targets of miR-92a-1-5p. However, in *in vitro* experiments we could only confirm the fructose transporter *Slc2a5* as a direct target of miR-92a-1-5p. Notably, in our transcriptome analysis, *Slc2a5* was the most downregulated gene in fasted BAT.

Furthermore, our data provide evidence that iBACs can take up fructose avidly and metabolize it, suggesting a physiological significance of fructose uptake by SLC2A5 in brown adipocytes. Previous studies have already shown that the supplementation of fructose to the growth medium of white adipocyte precursor cells can increase their differentiation capacity and that fructose is a potent lipogenic substrate, triggering the formation of oleate and palmitate in human mature white adipocytes (Varma et al., 2015a; 2015b). Moreover, it was recently shown that despite the low abundance of fructose in the systemic circulation in physiological conditions, intravenously administered labeled fructose can be detected in BAT of mice (Jang et al., 2018) and that *Slc2a5* is strongly induced in BAT of mildly cold-stressed mice (Sanchez-Gurmaches et al., 2018). However, further studies need to examine the fate of fructose, the regulatory role of fructose and/or products of fructose catabolism, and the implications of ChREBP signaling and BCAA metabolism in BAT during times of nutrient restriction.

Of note, our findings are derived from male mice only and a recent report showed systemic differences (mostly male hyperinsulinemia) in the fasting/feeding response in male and female C57Bl/6J mice (Bazhan et al., 2019). In this study BAT weight and the expression of genes measured in BAT was not different between males and females (Bazhan et al., 2019). As the functional axis we report here seems to act cell autonomous, it is possible that it also operates in females, although this needs to be formally tested.

Taken together, our study delineated the transcriptome and miRnome responses of BAT from mice that were challenged with an acute 24 h fasting bout and mild cold stress. Targeted *in vitro* experiments validated a novel, fasting-selective pathway involving p53 signaling to regulate the fructose transporter *Slc2a5* by miR-92a-1-5p modulation. Furthermore, our results add to data suggesting a metabolic role of fructose as an energy substrate in brown adipocytes (Figure 7).

Contribution to the Field Statement: Active BAT is a highly energy-consuming tissue and is of critical importance for the regulation of whole-body energy homeostasis. Thermogenic active depots found in adult humans, frame BAT as an attractive pharmacological target for the treatment of metabolic diseases. However, the regulation of thermogenic properties of BAT under fasting conditions, especially under simultaneous mild cold stress, has not been explored until now. We elucidated the transcriptional and miRNA signature in BAT of mildly cold-stressed, acutely fasted mice and identified a highly nutrient-dependent pathway involving the transcription factor p53, miR-92a-1-5p, and the fructose transporter *Slc2a5*. We propose that repression of *Slc2a5*, by miR-92a-1-5p, downstream of p53, could represent a mechanism for limiting the thermogenic properties of BAT during fasting.

## Methods

### Mouse Experiments

All animal studies were approved by the Austrian Ministry for Education, Science and Research (Vienna, Austria, BMWFW-66.010/0087-WF/V/3b/2017) and performed strictly according to its guidelines. Animals (C57BL/6J, 12 weeks of age) were housed in a temperature-controlled (22°C) environment with a 12:12 h light-dark cycle. For fasting experiments, food was withdrawn at 9:00 a.m. for 24 h. To prevent coprophagy, fasted and *ad libitum* chow diet-fed mice were single-housed on grid bottoms without nesting material during the intervention. For high-glucose feeding experiments, C57BL/6J mice were fed a high-glucose diet (Ssniff Spezialdiaeten GmbH, Soest, Germany, E15629-34) for 12 weeks (Huber et al., 2019). Mice were sacrificed by cervical dislocation, and harvested interscapular BAT depots were immediately frozen in liquid nitrogen.

### Metabolic Cages

Metabolic assessment of mice was performed using an indirect calorimetry system (TSE PhenoMaster, TSE Systems, Bad Homburg, Germany). The animals were single-housed at room temperature, a regular light-dark (12:12 h) cycle, and with free access to food and water. Mice were acclimated to the metabolic cages for 48 h before metabolic recording. After 1 week of metabolic recording, the fasting experiment was performed by withdrawing the food at 9:00 a.m. for 24 h. O_2_ consumption, CO_2_ production, and locomotor activity (using infrared sensor frames) were measured every 15 min.

### Cell Culture

#### iBACs

Immortalized brown preadipocytes [iBACs (Harms et al., 2014)] were a kind gift of Patrick Seale. iBACs were maintained in DMEM (Thermo Fisher Scientific, Waltham, MA, United States, 41966-029) containing 4.5 g/L glucose supplemented with 10% (v/v) heat-inactivated fetal bovine serum (FBS, HyClone TM, Thermo Fisher Scientific, Waltham, MA, United States, SV30160.03), 1% penicillin and streptomycin (Thermo Fisher Scientific, Waltham, MA, United States, 15140-122), and 20 mM HEPES (Thermo Fisher Scientific, Waltham, MA, United States, 1,560-080). Cells were cultivated in a humidified atmosphere of 5% CO_2_ and 95% air at 37°C. iBACs were induced to undergo adipogenesis at a confluence of ∼90% by the addition of 500 nM dexamethasone, 1 nM triiodothyronine, 0.5 mM 3-isobutyl-1-methylxanthine, 1 µg/ml insulin, and 125 mM indomethacin (all Sigma-Aldrich, St. Louis, MI, United States) to the growth medium. From day three on, the growth medium was supplemented with 1 µg/ml insulin and 1 nM triiodothyronine, and changed every other day. Fully differentiated iBACs (day 7–10) were used for the experiments.

### Stromal Vascular Fraction Isolation and Differentiation Into brown Adipocytes

BAT depots were harvested, finely minced with scissors, and incubated in collagenase solution (4 mg/ml collagenase Type II (Thermo Fisher Scientific, Waltham, MA, United States, 17101015), 10 mM CaCl_2_, 0.5% FFA-free BSA (Sigma-Aldrich, St. Louis, MI, United States, 126609) for ∼10 min at 37°C. Digestion was stopped by adding 30 ml of growth medium and cells were filtered through a 100 µm sieve. SVF was pelleted by centrifugation at 600 *x g* for 15 min. After adding 1 ml of erythrocyte lysis buffer and incubating for 1 min, 30 ml of growth medium was added and filtered through a 70 µm sieve. The SVF was pelleted by centrifugation at 600 *x g* for 15 min und seeded in T75 flasks in growth medium (DMEM/F12 with glutamax (Thermo Fisher Scientific, Waltham, MA, United States, 10565018), supplemented with 10% FBS, 1% penicillin and streptomycin). To induce differentiation, the growth medium was supplemented with 1 µM dexamethasone, 100 nM triiodothyronine, 0.5 mM 3-isobutyl-1-methylxanthine, 1.5 µg/ml insulin, and 1 µM rosiglitazone (all Sigma-Aldrich, St. Louis, MI, United States). After 3 days, medium was changed to growth medium supplemented with 1.5 µg/ml insulin and 100 nM triiodothyronine. Primary brown adipocytes were harvested 7–9 days after differentiation start.

### Treatments

For starvation experiments, cells were washed with PBS (Thermo Fisher Scientific, Waltham MA, United States, 10010-015) and maintained in starvation medium [HBSS (Thermo Fisher Scientific, Waltham, MA, United States, 14175-053) supplemented with 10 mM HEPES (Thermo Fisher Scientific, Waltham, MA, United States, 1560-080)] for 24 h. To pharmacologically stabilize p53, iBACs were treated with 1 µM Idasanutlin (Selleck Chemicals, Houston, United States, RG-7388) for 24 h. For fructose experiments, iBACs were incubated for 24 h in growth medium supplemented with 5 g/L fructose (Sigma-Adrich, St. Louis, MI, United States, F3510).

### Tissue Isolation

Tissues were homogenized in Qiazol (Qiagen, Hilden, Germany, 79306) using Magnalyser beads (PeqLab, Radnor, United States, 412-0201) at 6.500 rpm for 20 s and two runs with the TissueLyser (Qiagen, Hilden, Germany). Samples were cooled with short taps in N_2_ between the runs and incubated for 5 min at room temperature. RNA was isolated with PeqGOLD total RNA kit (Peqlab, Radnor, United States, 12-6634) according to the manuals. Sample purification and concentration was quantified with NanoDrop® ND-1000 (Peqlab, Radnor, United States). For western blotting experiments, tissues were homogenized with Magnalyser beads in radioimmunoprecipitation assay (RIPA) buffer (50 mM Tris-HCl, 150 mM NaCl, 2 mM EDTA, 50 mM NaF, 0.1% SDS, 0.5% Na-deoxycholate, 1% NP-40, adjusted to pH 7.2 — 7.4) supplemented with PIC (complete Tablets EASYpack, Roche, Basel, Switzeland, 04693116001) and PhosStop (Roche, Basel, Switzeland, 04906837001), incubated on ice for 20 min, and centrifuged at 15.000 *x g* for 15 min. The protein concentration of cleared supernatants was analyzed with a bicinchoninic acid assay kit (BCA, Thermo Fisher Scientific, Waltham, MAs, United States).

### qPCR Analysis

For qPCR analysis, isolated total RNA was reverse transcribed to cDNA by using High-Capacity cDNA Reverse Transcription Kit (Thermo Fisher Scientific, Waltham, MA, United States, 4368814) and amplified using Blue SybrGreen qPCR mastermix (Biozym Scientific, Olendorf, Germany, 331416XL). All primer sequences are listed in Table 1. TFIIb was used as reference gene.

**TABLE 1:** Nucleotide sequences for qPCR, cloning, and ChIP primers.

	Fwd (5′–3′)	Rev (5′–3′)
Ucp1	GGA​TTG​GCC​TCT​ACG​ACT​CA	TAA​GCC​GGC​TGA​GAT​CTT​GT
Pgc1a	GGT​CAA​GAT​CAA​GGT​CCC​CA	TCA​TAG​CTG​TCG​TAC​CTG​GG
Cidea	ATC​ACA​ACT​GGC​CTG​GTT​ACG	TAC​TAC​CCG​GTG​TCC​ATT​TCT
Prdm16	CAG​CAC​GGT​GAA​GCC​ATT​C	GCGTGCATTCGCTTGTG
Dio2	GTC​CGC​AAA​TGA​CCC​CTT​T	CCC​ACC​CAC​TCT​CTG​ACT​TTC
Trp53	ACA​TGA​CGG​AGG​TCG​TGA​G	AAT​TTC​CTT​CCA​CCC​GGA​TA
Cdkn1a	CCT​GGT​GAT​GTC​CGA​CCT​G	CCA​TGA​GCG​CAT​CGC​AAT​C
Mdm2	AGC​AGC​GAG​TCC​ACA​GAG​AC	ATC​CTG​ATC​CAG​GCA​ATC​AC
Gadd45a	CCG​AAA​GGA​TGG​ACA​CGG​TG	TTA​TCG​GGG​TCT​ACG​TTG​AGC
Ddit4	TCT​TCG​CTG​ACC​GCG​CTA​GC	CGG​CCG​GAG​TTC​GAG​ACG​AG
Slc2a5	GCT​GCA​GCC​AAA​TTG​CCC​AAT​CG	CGG​GGC​CAG​CTC​CCC​TAA​GT
Fasn	CAC​CAA​TAC​AGA​TGG​CAG​CA	CCA​GCT​GGC​TGA​TAC​AGA​GA
Ppara	GCG​TAC​GGC​AAT​GGC​TTT​AT	GAA​CGG​CTT​CCT​CAG​GTT​CTT
Gamt	GCA​GCC​ACA​TAA​GGT​TGT​TCC	CTC​TTC​AGA​CAG​CGG​GTA​CG
Srebf1	AAG​CCA​ATC​ACT​GAA​GGA​CCT​GG	AAA​GAC​AAG​GGG​CTA​CTC​TGG​GAG
Elovl6	CGT​AGC​GAC​TCC​GAA​GAT​CAG​CC	AGC​GTA​CAG​CGC​AGA​AAA​CAG​GA
Glut4	GGC​ATC​AAT​GCT​GTT​TTC​TAC	GCTGGAACCGCTTCCAGC
Sesn1	CGG​ACC​AAG​CAG​GTT​CAT​CC	TGA​TGT​TAT​CCA​GAC​GAC​CCA​AA
Mid1ip1	GGT GAA CAA CAT GGA CCA GA	CGC TGA CCT CGT CTA TCT CC
Khk-a	TGG​ACT​TAC​GAT​ATG​TCC​TT	GCC​TCG​TTG​ATG​ATG​ACT​GTA​G
Chrebp-b	TCTGCA GATCGCGTGGAG	CTT GTCCCGGCATAGCAAC
TFIIb	GTC​ACA​TGT​CCG​AAT​CAT​CCA	TCA​ATA​ACT​CGG​TCC​CCT​ACA​A
	CLONING PRIMER	
Slc2a5 insert	TTG​CTC​GAG​CAC​AGC​CAT​CTT	TGG​CGG​CCG​CCA​GAA​TGT​GCT​T
	ChIP	
miR-92a-1-5p	TTG​GGA​TTT​GTC​GCA​ATG​CTG	TCT​GGT​CAC​AAT​CCC​CAC​CA
p21	CTG​TTG​CCT​CTC​GGA​GAC​C	CCT​GAA​GGC​CAG​AAA​GCT​AGT
Neg. Ctrl	TGA​GCA​CAG​GAG​AAA​AGG​CAA	GCC​TAC​CAA​GAC​AAA​TGA​GCA​G

### Western Blot Analysis

Immunoblotting was performed as described elsewhere (Prokesch et al., 2016). WES digital western blot (Bio-techne, Proteinsimple, Minneapolis, and Minnesota) was performed according to the manufacturer’s guidelines, using 10 µg of protein.

Antibodies used: p53 (D2H9O, Cell Signalling, Danvers, MA, United States, 32532), GAPDH (Cell Signalling, Danvers, MA, United States, 2118S), β-actin (Abcam, Cambridge, United Kingdom, ab6276).

### miRNA Isolation

miRNA isolation from iBACs and BAT was performed with the Qiagen miRNeasy Kit (Qiagen, Hilden, Germany, 217004) according to the manufacturer’s guidelines. In short, about 100 mg of BAT was dissected in 700 µl Qiazol (Qiagen, Hilden, Germany, 79306) as described above. 140 µl of chloroform was added to the samples, shaked vigorously and incubated for 2–3 min at room temperature. After spinning the samples at 12.200 rpm for 15 min at 4°C, the supernatant was mixed with 1.5 volume of 100% ethanol. A maximum of 500 µl was loaded to the MiniElute tubes and the protocol was processed as outlined by the manufacturer’s instructions and total RNA was eluted in 20 µl EB-buffer (Qiagen, Hilden, Germany). All samples were quality checked on a BioAnalyzer BA2100 station (Agilent, Foster City, CA, United States).

### miRNA-Reverse Transcription and miRNA-qPCR

Reverse transcription (miRCURY LNA RT Kit, Qiagen, Hilden, Germany, 339340) and qPCR (miRCURY LNA SYBR Green PCR Kit, Qiagen, Hilden, Germany, 339345) of miRNAs was performed according to the manufacturer’s guidelines.

### Transcriptome Analysis and miRNA Sequencing

For whole Transcriptome analysis 200 ng of total RNA were used with the GeneChip™ Human Transcriptome Assay 2.0 kit (Thermo Fisher Scientific, Waltham, MA, United States) according to manufacturer’s instructions. Arrays were washed after hybridization on a GeneChip™ Fluidics Station 450 and scanned on a GeneChip™ Scanner 3,000 7G. Raw microarray data have been submitted to Gene Expression Omnibus (GEO accession number GSE199963).

For miRNA library preparation 100 ng of total RNA were used with the NEBNext® Small RNA Library Prep Set for Illumina® (New England Biolabs, Ipswich, MA, United States, E73305) according to manufacturer’s instructions. Quality of libraries was checked on an Agilent BioAnalyzer BA2100 station, pooled and sequenced in an Illumina HiSeq lane (Illumina, Eindhoven, Netherlands). FastQ raw data are publicly available in the European Nucleotide Archive (ENA) with the accession number PRJEB51729 (https://www.ebi.ac.uk/ena, last access March 2022).

Transcriptome and miRNA raw data were normalized and analyzed using Partek^®^ Genomics Suite^®^ Software 6.6 (Partek Incorporated, St. Louis, MI, United States) according to standard settings. Array data were normalized using robust multi-chip average normalization (RMA).

Gene Set Enrichment Analysis (GSEA) was performed with the GSEA app (Broad Institute) on a list ranked according to expression changes between BAT of fasted and fed mice. Hallmark analysis was conducted according to default settings and top five enriched and de-enriched hallmark pathways were identified and displayed (Subramanian et al., 2005).

Putative targets of mmu-miR-92a-1-5p (miRBase accession number MIMAT0017066) were obtained from miRWalk 2.0 (Dweep et al., 2011; Sticht et al., 2018), integrating the output of several miRNA-target prediction algorithms. The query for potential mmu-miR-92a-1-5p MREs was limited to 3′UTRs of mRNAs. The interaction of mmu-miR-92a-1-5p and *Slc2a5* was predicted by rna22 (Miranda et al., 2006) and RNAhybrid (Rehmsmeier et al., 2004).

### Glycolysis Stress Test

iBACs were detached at day 5 of differentiation with 0.5 mg/ml collagenase P (Sigma-Aldrich, St. Louis, MI, United States, 11213857001) and 2.5% Trypsin (Thermo Fisher Scientific, Waltham, MA, United States, 15400054) in PBS and seeded at a density of 5 x 10^4 cells per well in a seahorse 96-well plate (Agilent, Santa Clara, CA, United States. After 24 h, a glycolysis stress test was performed, according to manufacturer’s instructions (Agilent, Santa Clara, CA, United States). The extracellular acidification rate (ECAR) was measured using XF96 Extracellular Flux Analyzer (Agilent, Santa Clara, CA, United States) before and after acute injection of 5 g/L fructose or glucose (Sigma-Aldrich, St. Louis, MI, United States, 50-99-7), 1 µM oligomycin-A or 50 mM 2-deoxy-glucose.

### Nuclear Magnetic Resonance Metabolomics

NMR metabolomics was performed as previously published (Alkan et al., 2018). In short, iBACs were treated with growth medium supplemented with 5 g/L Fructose for 24 h iBACs were washed extensively with PBS and harvested for NMR metabolomics as described. Metabolites were extracted using methanol, NMR spectra were recorded and processed in Matlab 2014a to obtain aligned and normalized datasets.

### Luciferase Assay

For Luciferase assay, the 3′UTR of *Slc2a5* harbouring predicted seed matches for miR-92a-1-5p was cloned in a PsiCheck2 vector (Promega, Madison, WI, United States, C8021). HEK293 cells were transfected with 1 µM mmu-miR-92-1-5p-mimic (Horizon discoveries, Waterbeach, United Kingdom, MIMAT0017066) or non-targeting control (Horizon, CN-001000-01–05) and 0.2 µg PsiCheck2 vector using lipofectamine 3,000 (Thermo Fisher Scientific, Waltham, MA, United States, as transfection reagent. The medium was changed after 24 h of transfection and luciferase assay was performed on day 2 after transfection. The luciferase assay was performed according to the manufacturer’s instructions (Thermo Fisher Scientific, Waltham, MA, United States, 16185).

### ChIP-qPCR

ChIP-qPCR was performed according to an established protocol (Prokesch et al., 2016). In short, fully differentiated iBACs were crosslinked with 1% formaldehyde (Thermo Fisher Scientific, Waltham, MA, United States) for 15 min at RT. Crosslinking was stopped by adding 125 mM glycin to the medium for 5 min. Afterwards, chromatin was sonicated in Bioruptor^®^ Pico Microtubes (Diogenode, Denville, United States, C30010016) for 10 cycles (30 s on/30 s off) by using the Diogenode Bioruptor (Diogenode, Denville, United States, B01020001) and fragment size was analyzed by running the DNA/Chromatin fragments on an agarose gel. Sonicated samples were washed twice according to the protocol. IP was performed using precleared Protein G DynaBeads magnetic beads (Thermo Fisher Scientific, Waltham, MA, United States, 10003D). 1.25 µg of the following antibodies was used: a-p53 (D2H9O, Cell Signalling, Danvers, MA, United States, 32532), a-IgG (Santa Cruz, Santa Cruz, United States, sc-2027). Immunoprecipitated chromatin and input chromatin were reverse cross-linked and column purified. DNA was subjected to SYBR green qPCR. Primers designed at loci without p53 binding sites served as negative control. All primer sequences are listed in Table 1.

### Histology

Immunohistochemical staining of formalin-fixed, paraffin-embedded BAT depots was performed after antigen retrieval (93°C, 15 min at pH 6) and peroxidase blocking (Agilent, Foster City, CA, United States, S202386-2) using the UltraVision LP detection system (Thermo Fisher Scientific, Waltham, MA, United States, 12643997) according to the manual with UCP1-antibody (1.25 µg/ml; Abcam, MA, United Kingdom, 10983). AEC (3-amino-9-ethyl carbazole) chromogen (Thermo Fisher Scientific, Waltham, MA, United States, 001122) was used for color detection. Counterstaining with hematoxylin was done on all slides. Hematoxylin and eosin stainings were quantified by using ImageJ Brown adipocyte area was indicated as square pixels.

### Statistical Analysis

If not stated otherwise, all experiments were performed at least three times independently. Statistical analysis was performed using GraphPad Prism 8 (GraphPad Software). Statistically significant differences were determined as described in the figure legend. If not noted otherwise, data are represented as mean values ± SEM with the following levels of statistical significance: *p < 0.05, **p < 0.01, and ***p < 0.001. Differences not indicated with asterisks or indicated with ns are not statistically significant (*p* > 0.05).

## Data Availability

The data presented in the study are deposited in the NCBI GEO repository, accession number GSE199963 (transcriptome data) and the EBI ENA repository, accession number PRJEB51729 (miRNA sequencing) https://www.ebi.ac.uk/ena, PRJEB51729; https://www.ncbi.nlm.nih.gov/geo/, GSE199963.
